# Comparative studies on physicochemical properties, volatile flavor substances and microbial community of Mianning ham at different altitudes

**DOI:** 10.3389/fmicb.2025.1536749

**Published:** 2025-02-10

**Authors:** Qianhui Zeng, Lili Ji, Wei Wang, Jiamin Zhang, Ting Bai, Ling Gan, Lin Chen

**Affiliations:** ^1^Key Laboratory of Meat Processing of Sichuan Province, Chengdu University, Chengdu, China; ^2^College of Veterinary Medicine, Southwest University, Chongqing, China

**Keywords:** Mianning ham, altitudes, physicochemical properties, volatile components, microbial community

## Abstract

**Background:**

This study explores the impact of varying altitudes on the quality characteristics of Mianning ham.

**Methods:**

By utilizing Solid-Phase Microextraction—Gas Chromatography–Mass Spectrometry (SPME-GC-MS) technology and high-throughput sequencing techniques, the physicochemical properties, volatile flavor compounds, and shifts in microbial communities of Mianning ham at different altitudes were investigated.

**Results:**

Ham’s water content, aw, pH, malondialdehyde content,and nitrite content at high altitudes were higher, while the salt content of ham at low altitudes was higher. 112 volatile compounds were identified in ham fermented for 0, 1, and 2 years at low altitude and high altitude, and the volatile compounds in ham at high altitude were more abundant than those in ham at low altitude. The main flavor compounds were 1-octene-3-ol, Nonyl aldehyde, Octanal, and 15 other volatile compounds. At the phylum level, the dominant bacteria were Firmicutes and Proteobacteria, and the fungus was Basidiomycota. *Staphylococcus* was the dominant bacterium at the genus level, and *Aspergillus* was the dominant fungus.The correlation analysis of microorganisms and volatile flavor substances showed that *Cobetia* promoted the formation of Benzaldehyde in ham at low altitudes. In contrast, *Kocuria* promoted the formation of 1-Octanol, Heptanol,1-Butanol, 2-Heptanone, 3-Hydroxy-2-butanone, Octanal, and Hexanal in ham at high altitudes.

**Discussion:**

There were obvious differences in the quality of Mianning ham between the two altitudes.

## 1 Introduction

The fermentation process of ham is a complex biochemical transformation involving the hydrolysis and oxidation of muscle proteins and fats and metabolic reactions by microorganisms (Morgan and Breen, 2021). Relevant research indicates that the moisture content and water activity of dry-cured ham progressively decrease with the extension of the processing time (Marušić Radovčić et al., 2021). During fermentation, changes in the content of free amino acids are related to the degree of protein degradation, and these amino acids directly impact the ham flavor (Zhou et al., 2020). Fatty acid oxidation in ham is one of the critical pathways for flavor formation. Analytical studies have found that free fatty acids (FFA) in mature ham change during the ripening process, which is closely related to lipid oxidation and significantly impacts ham flavor development (Li et al., 2022). The study of Xuanwei ham found that the flavor substances of hams from different years are distinctive, which may be related to the metabolic activities of specific microorganisms during the maturation process (Li et al., 2024). The color affects the ham’s visual appeal and is closely related to its flavor characteristics (Guo et al., 2021). In recent years, researchers have quickly distinguished the appearance, texture, and other properties of hams of different quality levels based on sensory descriptive analysis combined with rapid instrumental measurements (Barbieri et al., 2016). Microorganisms play a crucial role in the processing and ripening of ham. Studies have shown that the microbial community structure of ham from different regions is also significantly different due to the differences in the environment and processing technology. Yunnan Sanchuan ham undergoes various dynamic changes in its microorganisms count on both the surface and inside during processing, and staphylococci, pseudomonas, and mold play crucial roles at different stages (Qin et al., 2024).

Mianning ham is a specialty of Mianning County in Sichuan Province, renowned for its unique flavor and production craftsmanship. It is not only a traditional meat product of the local area but also has been certified with the national geographical indication for agricultural products. Mianning County is located in the low latitude and high altitude area, and it belongs to the subtropical monsoon climate as a whole, with some regions having plateau climate characteristics. Therefore, the unique geographical environment is conducive to ham’s curing and natural fermentation. Altitude is one of the many environmental factors affecting agricultural product quality (Chen et al., 2023). Environmental differences such as temperature, humidity, and UV intensity brought about by different altitudes profoundly impact the growth and quality of crops (Ali et al., 2021). Particular temperature and humidity conditions will promote moderate hydrolysis of proteins in meat products to improve the taste of meat products and the stabilization of myoglobin in meat products to improve the color of meat products (Gregory, 2010). Research has shown that the nutritional composition of yak meat is related to the local altitude, and yaks from high-altitude areas are characterized by high protein, low fat, and rich nutrition (Xin et al., 2022). For ham, climatic factors such as temperature and humidity in regions with varying altitudes can significantly influence the drying and maturation processes of the product.

Studies on the differences in the quality characteristics of Mianning ham at different altitudes have yet to be reported. This study aims to reveal the specific differences in the quality characteristics of Mianning ham at different altitudes by analyzing the changes in physical and chemical properties, volatile flavor substances, and microbial communities of Mianning ham at different altitudes. The results of this study will not only provide a scientific basis for the processing and quality control of Mianning ham but also provide a reference for the production of ham at other altitudes and, at the same time, provide theoretical support for the development of the ham industry and the protection of geographical indications products.

## 2 Materials and methods

### 2.1 Sample preparation

In this study, the hind legs of pigs crossed between Liangshan Wujin pigs and breeders Changbai or York were selected as samples. Hybrid pigs are typically fed rice, buckwheat, and green feed. After 180 days of feeding, they can be slaughtered when their live weight reaches 100 kg. Select pig hind legs that weigh around 8–10 kg on average, have more lean meat, and are less fat for curing. Adopt the particular process flow for Mianning ham, including disk legging, trimming, curing, washing, fermentation, and storage. A total of 12 pig hind legs that had been fermented for 6 months (0 year), 1 year, and 2 years at altitudes of 1,600 and 2,544 m, respectively, were selected. After vacuum packaging in the field, samples are transported back to the laboratory at low temperatures and stored at −20°C.

### 2.2 Measurement of physical and chemical properties

After homogenizing the samples, the water content, water activity, pH, malondialdehyde, nitrite, and chloride were determined. Water content was determined by CS-120H moisture tester (Shenzhen, Guanya, China), water activity meter GYW-1 (Shenzhen, Guanya, China), and pH was determined by testo206pH1 (Germany). According to Zhu et al. (2022), the content of malondialdehyde was determined by high-performance liquid chromatography. Spectrophotometry described by Iacumin et al. (2019) determined the content of nitrite. The chloride content was measured using the Perez-Palacios et al. (2022) method with a slight modification. Chromaticity values were determined according to the method described by Pathare et al. (2013) with a slight modification using a CR-400 portable colorimeter (Konica Minolta Co., Ltd., Tokyo, Japan), using a standard color plate for whiteboard correction. Luminance (L*-value), redness (a*-value), and yellowness (b*-value) were recorded. All indicators were repeated three times.

### 2.3 Determination of flavor substances

Headspace solid-phase microextraction (HS-SPME) was used to extract the flavor substances from Mianning ham. Mianning ham samples from different altitudes and different fermentation years were minced separately, and 3.00 g was accurately weighed in a 15 mL headspace vial, to which 1 μ2,4,6-trimethylpyridine was added as an internal standard and sealed with a cap. The conditions of the CTC autosampler were as follows: heating box at 75°C, heating for 45 min, sample extraction for 20 min, and resolving for 5 min. Flavor substances were analyzed and identified by using a gas chromatography-mass spectrometer (GC–MS) was used to analyze and identify the flavor substances. GC conditions:HP-5MS Chromatographic column (30 m × 25 mm, 0.25 μm); the pressure was 32.0 kPa; the flow rate was 1.0 mL/min; the carrier gas was helium, and the sample was injected without shunt; the temperature of the inlet port was 250°C. The column temperature ramp-up program: the starting temperature was 40°C and kept for 15 min; the temperature was increased to 160°C at 3°C/min and kept for 0 min; the temperature was increased to 230°C at 4°C/min and kept for 5 min. MS conditions: electron ionization source (EI); electron energy 70 eV; ion source temperature 230°C, quadrupole temperature 150°C; mass scanning range: 40–500 m/z; detector voltage 350 V.

The chromatogram of the obtained sample was integrated, and the volatile compounds corresponding to the peaks on the matching chromatogram were retrieved in the NIST 14 L spectrum library under the condition of 80% matching degree. For further confirmation, the Kovats index (KI) was calculated using a mixture of n-alkanes (C5-C25).

### 2.4 High-throughput sequencing

In a sterile environment, a scalpel cut pieces of meat with a thickness of about 2 cm inside each Mianningn ham, which was marked and immediately placed in sterile freezing tubes and stored at −80°C temperature. Sample DNA purification: Zymo Research BIOMICS DNA Microprep Kit was used for sample gDNA purification. gDNA integrity was detected by 0.8% agarose electrophoresis, followed by nucleic acid concentration detection (PicoGreen dye method) using Tecan F200. PCR amplification: According to the sequencing region, specific primers with the index sequence were synthesized to amplify the 16S rDNA V4 region of the sample. Specific primers with index sequences to amplify the 16S rDNA V4 region of the samples and the sequences of the amplification primers were as follows: Primer5′-3:515F (5′-GTGYCAGCMGCCGCGGGTAA-3′) and 806R (5′-GGACTACHVGGGTWTCTAAT-3′) PCR enzyme: TOYOBO KOD-Plus-Neo DNA Polymerase (KOD-401B) PCR instrument: Applied Biosystems® PCR PCR program: pre-denaturation at 94°C for 1 min, 1 cycle; denaturation at 94°C for 20 s, annealing at 54°C for 30 s and extension at 72°C for 30 s, 25–30 cycles; 72°C for 5 min, 1 cycle; and 4°C holding. Each sample was subjected to 3 PCR technical replicates, and an equal amount of linear phase PCR products was taken and mixed for subsequent library construction. PCR product detection, purification, and quantification: PCR products were mixed with a 6-fold up-sampling buffer, followed by electrophoretic detection of the target fragments using a 2% agarose gel. Samples that passed the test were recovered from the target bands using Zymoclean Gel Recovery Kit (D4008), quantified using Qubit@ 2.0 Fluorometer (Thermo Scientific), and finally mixed in equimolar quantities. Library construction: NEBNext Ultra II DNA Library Prep Kit for Illumina (NEBE7645L) from NEW ENGLAND BioLabs was used for library construction. High-throughput sequencing: PE250 sequencing was used, and the sequencing kit was NovaSeq 6000 SP Reagent Kit V1.5 for Illumina.

### 2.5 Data analysis

Excel 2021 was used for statistical data processing, SPSS Statistics27.0 (IBM, Chicago, USA) for analysis of variance (ANOVA), Origin 2021 for plotting principal component analysis plots (PCA plots), histograms, and heat clustering plots, and R language for drawing heat clustering plots. All experiments were conducted three times independently, and the results were expressed as mean ± standard deviation (SD).

## 3 Results and analysis

### 3.1 Analysis of physical and chemical results

Table 1 shows the results of physicochemical indexes of Mianning hams from different fermentation years at low and high altitudes. MP moisture content (43.16–62.46 g/100 g) was significantly higher (*p* < 0.05) than MY moisture content (38.99–60.86 g/100 g). This may be because high-altitude areas usually have cold climates and high humidity, which are favorable for moisture retention in hams. The moisture content of hams from different years at the same elevation was decreasing from year to year, a result that is consistent with the findings of Chen et al. (2024), who found that the moisture content of Sanchuan hams also showed a gradual decrease during the fermentation process. The difference in moisture content between the 0 year hams and the 1 and 2 year hams was significant (*p* < 0.05). During the curing process of hams, the water in the hams evaporated with the increase of fermentation time. The addition of salt led to water penetration inside the hams to the outside. This penetration continued with the increase of fermentation time, which further reduced the moisture content of the hams (Martuscelli et al., 2017).

**Table 1:** Physical and chemical indexes of Mianning ham at different altitudes.

Physicochemical index	MY			MP		
	MY0	MY1	MY2	MP0	MP1	MP2
Moisture content (g/100 g)	60.86 ± 0.36^Ab^	39.43 ± 2.72^Bb^	38.99 ± 0.86^Bb^	62.46 ± 3.14^Aa^	44.93 ± 0.69^Ba^	43.16 ± 0.63^Ba^
aw	0.78 ± 0.02^Ab^	0.77 ± 0.03^Ab^	0.74 ± 0.00^Ab^	0.85 ± 0.02^Aa^	0.82 ± 0.01^Ba^	0.81 ± 0.01^Ba^
pH	5.29 ± 0.01^Ca^	5.34 ± 0.01^Ba^	5.40 ± 0.02^Ab^	5.32 ± 0.03^Ca^	5.36 ± 0.02^Ba^	5.73 ± 0.02^Aa^
Malondialdehyde (mg/kg)	0.47 ± 0.02^Cb^	0.66 ± 0.03^Bb^	0.92 ± 0.03^Aa^	0.64 ± 0.04^Ca^	0.82 ± 0.0^3Ba^	1.10 ± 0.04^Aa^
Nitrite (mg/kg)	0.67 ± 0.05^Cb^	0.72 ± 0.12^Bb^	0.94 ± 0.03^Ab^	0.96 ± 0.06^Ba^	1.08 ± 0.09^Aa^	1.12 ± 0.14^Aa^
Chloride (%)	5.56 ± 0.04^Ca^	5.82 ± 0.02^Ba^	6.71 ± 0.02^Aa^	5.29 ± 0.02^Cb^	5.57 ± 0.03^Bb^	6.40 ± 0.02^Ab^
L*	43.86 ± 0.02^Aa^	37.12 ± 0.89^Ba^	34.15 ± 0.66^Ca^	38.03 ± 0.88^Ab^	35.64 ± 0.45^Ab^	32.84 ± 0.77^Ab^
a*	8.06 ± 0.48^Ca^	10.64 ± 0.58^Ba^	13.36 ± 0.28^Ab^	7.11 ± 0.47^Ca^	9.86 ± 0.24^Ba^	12.93 ± 0.74^Aa^
b*	4.09 ± 0.20^Cb^	5.91 ± 0.18^Bb^	8.32 ± 0.63^Aa^	5.97 ± 0.7^1Ca^	7.09 ± 0.70^Ba^	8.68 ± 0.81^Aa^

MY represents low-altitude ham (1,600 m above sea level), MP represents high-altitude ham (2,544 m above sea level), MY0, MY1, and MY2 denote Mianning hams fermented for 0, 1, and 2 years at low altitude, respectively, and MP0, MP1, and MP3 denote coronation hams fermented for 0, 1, and 2 years at high altitude, respectively. Different uppercase letters (A, B, C) denote the variation between different years at the same altitude (*p* < 0.05), and different lowercase letters (a, b) denote the variation between different altitudes at the same year (*p* < 0.05).

aw is a parameter that describes the state of water-solute binding in food and indicates the degree to which the water is available or can be utilized by microorganisms (Mathlouthi, 2001). MP water activity was significantly higher than MY (*p* < 0.05). High elevations are usually calmer, and the air humidity is different from lower elevations; low temperatures can slow the evaporation of water, and the unique humidity conditions may help to retain water in the ham, thus affecting water activity. With the extension of processing time, the aw is decreasing continuously. Studies have shown that when the general aw is higher than 0.8, ham is susceptible to microbial contamination (Olaimat et al., 2020), so reducing aw is conducive to preserving ham.

There was no significant difference in pH value between 0 and 1 year ham at the two elevations. The pH value increased with the extension of fermentation time, and the pH value of ham at the same altitude showed a significant difference between different years (*p* < 0.05), and the pH value of ham in the second year was the highest, reaching 5.32 and 5.73, respectively, which was different from the result of Li et al. (2024a) in Xuanwei ham. They found that Xuanwei ham fermented for 2 years had a pH of 5.95.

Malondialdehyde is a small molecule compound produced in living organisms by lipid peroxidation, a secondary lipid oxidation product (Zamora et al., 2024). MP0 and MP1 malondialdehyde contents were significantly higher than MY0 and MY1 malondialdehyde contents (*p* < 0.05), and there was no significant difference in malondialdehyde content of hams fermented for 2 years at different altitudes. Increased production of reactive oxygen and nitrogen species (RONS) due to oxidative/reductive stress at high altitudes due to reduced oxygen pressure may increase oxidative damage to lipids, proteins, and DNA (Gaur et al., 2021). At the same altitude, with the increase of fermentation time, the content of malondialdehyde in ham of three fermentation years was significantly different (*p* < 0.05). The ham fermented for 2 years had the highest degree of oxidation. This result is in agreement with the study of Andrés et al. (2004), who found that the degree of lipid oxidation of Iberian hams varied under different salt contents and processing conditions, with a significant increase in the level of lipid oxidation with the increase in processing time, especially in hams processed with higher salt contents.

Nitrites are essential in forming color and flavor in dry-cured hams, but excessive residues pose a carcinogenic risk (Shakil et al., 2022). The study found that due to the specific environmental conditions at high altitudes, such as temperature, humidity, and soil properties, may affect microbial activity and nitrate transformation processes (Zhao et al., 2024). With the increase in fermentation years, the nitrite content in MY ham was significantly different (*p* < 0.05), and the nitrite content in MY2 was the highest, reaching 0.94 mg/kg. There was no significant difference between MP1 and MP2 ham.

The chloride content of MY was significantly higher than that of MP (*p* < 0.05). It may be that higher altitudes may have more favorable storage conditions, such as lower temperatures, that help keep the moisture content of the ham stable, which may reduce the use of salt (Garcia-Gil et al., 2014). Relevant studies have found that the level of salt content of ham is related to pH value. The lower the salt content, the higher the pH value of ham and the stronger the water retention capacity, which is consistent with the results of this study (Lin et al., 2020). With the extension of fermentation time, the salt content of MY and MP ham in different fermentation years showed a significant increase trend, and the salt content in the second year was the highest, reaching 6.71 and 6.40%, respectively.

Color values L*, a*, and b* are very important indicators in the evaluation of meat products such as ham, which can help evaluate ham’s maturity, freshness and quality (Tapp et al., 2011). Higher ambient temperature will accelerate the color transformation and corruption of meat. In comparison, lower ambient temperature and fast airflow can promote the formation of ferrimyoglobin and accelerate meat browning (Mancini and Hunt, 2005). MY brightness value L* was significantly higher than MP (*p* < 0.05), and at the same altitude, L* continued to decrease with the extension of fermentation year, which was consistent with the study of Li et al. (2024a), in which L* of ham fermented in year 0 was the highest. The redness value has a great relationship with the air drying process of ham (Andres et al., 2005). There was no significant difference in a* between 0 and 1 year fermented ham at two altitudes, but a* in MY2 was significantly higher than MP2 (*p* < 0.05). The b* of MP0 and MP1 was significantly higher than that of MY0 and MY1 (*p* < 0.05). The a* and b* of ham at the same altitude significantly differed with the increase in processing year (*p* < 0.05). It was found that myoglobin in ham would oxidize with the extension of maturation time, thus affecting the redness value of ham (Guo et al., 2021).

### 3.2 Analysis of volatile flavor substances

The contents of volatile flavor substances in the two elevations were shown in Table 2, and a total of 112 volatile compounds were identified. The results showed that a total of 65 volatile compounds were detected in MY, including 11 alcohols, 9 ketones, 9 aldehydes, 8 acids, 6 esters, 13 hydrocarbons, and 9 other types; 91 volatile compounds were detected in MP, including 18 alcohols, 13 ketones, 13 aldehydes, 2 acids, 7 esters, 20 hydrocarbons, and 9 other types. The volatile compounds of ham at high altitude are more abundant than those at low altitude.

**Table 2:** Volatile compounds content of Mianning ham at different altitudes (μg/kg).

Number	Compound name	KI	MY			MP		
			MY0	MY1	MY2	MP0	MP1	MP2
Alcohols								
1	Hexyl alcohol	867	60.93 ± 10.53^b^	29.88 ± 2.03^a^	—	172.18 ± 15.46^Aa^	19.20 ± 5.72^Cb^	50.97 ± 19.39^B^
2	3-Methyl-1-butanol	737	164.38 ± 20.65^b^	—	—	425.96 ± 66.76^a^	—	57.70 ± 10.19
3	Phenethyl alcohol	1,116	23.75 ± 1.25^b^	—	—	39.35 ± 7.51^a^	—	197.79 ± 78.15
4	1-Octen-3-ol	986	170.85 ± 7.99^Ab^	43.31 ± 7.17^Ba^	11.687 ± 2.166^Cb^	309.656 ± 115.59^Aa^	44.10 ± 1.42^Ba^	16.201 ± 1.43^Ca^
5	1-Heptanol, 5-methyl-	772	—	—	—	19.05 ± 7.28	—	—
6	DL-1-Phenethylalcohol;	1,066	—	—	—	40.21 ± 5.31	—	—
7	Capryl alcohol	1,068	55.49 ± 3.53^b^	—	—	73.52 ± 2.04^a^	—	—
8	1,5-Heptadiene-3,4-diol,2-methyl	681	—	—	—	9.71 ± 1.25	—	—
9	1-Pentanol	779	35.50 ± 3.08^b^	—	—	56.58 ± 35.10^a^	—	—
10	(E)-Oct-2-en-1-ol	1,067	16.55 ± 0.79^b^	—	—	77.12 ± 21.40^a^	—	—
11	2-[(2-furan methyl) amino] -2-methyl-1-propanol	768	—	—	—	5.02 ± 0.38	—	—
12	1-Heptanol, 5-methyl-,(5S)-	640	—	—	—	17.23 ± 1.71	—	—
13	1,6-Heptadien-4-ol,4-methyl-	712	—	—	—	10.77 ± 5.04	—	—
14	1-Hydroxy	975	11.77 ± 1.70^b^	—	—	25.96 ± 4.12^a^	—	—
15	1-Hexanol, 3-methyl-	1,053	—	—	—	10.708 ± 2.532	—	—
16	S,S)-2,3-Butanediol;	835	9.55 ± 1.04	—	—	—	—	55.76 ± 9.43
17	2,3-Butanediol	910	—	—	—	—	—	323.63 ± 40.39
18	1,2-Ethanediol,1,2-diphenyl-,	749	—	—	—	—	—	11.59 ± 1.28
19	3-Ethyl-4-nonanol	826	45.14 ± 0.91	—	—	—	—	—
20	1-Butanol	656	—	208.67 ± 16.08	—	—	—	—
Ketones								
21	2-Nonanone	1,092	13.12 ± 2.23^Cb^	34.48 ± 2.37^Bb^	98.25 ± 6.88^Ab^	18.86 ± 4.13^Ca^	64.72 ± 5.07^Ba^	102.79 ± 12.29^Aa^
22	3-Hydroxy-2-butanone	851	65.96 ± 0.76^b^	—	—	85.10 ± 3.44^a^	—	—
23	2-Heptanone	1,004	4.39 ± 6.33^Cb^	377.30 ± 5.75^Ab^	265.33 ± 4.21^Bb^	7.29 ± 1.06^Cb^	423.17 ± 105.39^Aa^	366.87 ± 194.55^Bb^
24	8-Nonen-2-one	1,009	—	7.91 ± 0.69^b^	—	—	250.65 ± 131.63^a^	214.70 ± 64.52
25	2,2,5-Trimethylhexane-3,4-dione	896	—	7.50 ± 1.62^b^	7.24 ± 1.73^b^	—	11.47 ± 3.48^a^	36.45 ± 5.34^a^
26	2,2,3-Trimethylcyclobutanone	718	—	—	—	—	—	7.235 ± 0.367
27	Acetone	653	344.33 ± 29.61^a^	—	6.45 ± 0.95^b^	119.23 ± 20.04^b^	—	87.49 ± 1.16^a^
28	3-Hexanone	719	—	—	—	8.02 ± 1.75	—	—
29	2-Pentanone,3,3-dimethyl	925	—	—	—	94.02 ± 2.16	—	—
30	2,3-Octadione	986	101.03 ± 7.85^a^	—	—	81.80 ± 45.61^b^	—	—
31	1-(2-Methylcyclopropyl)ethyl ketone	839	—	—	—	25.69 ± 2.03	—	—
32	3-Octanone;	980	—	—	—	15.57 ± 2.86	—	—
33	2-Butanone	602	—	—	—	7.31 ± 0.66	—	—
34	2(3H)-Furanone,3-aminodihydro-(8CI,9CI)	654	—		—	25.34 ± 4.67	—	—
35	2,4-Dimethyl-3-hexanone	892	—	4.80 ± 0.61^b^	—	—	12.17 ± 1.86^a^	36.55 ± 2.35
36	2-Octanone	900	—	—	—	—	26.62 ± 2.03	63.99 ± 10.54
37	1-Octen-3-one		—	—	—	—	2.85 ± 0.21	—
38	Hydroxy-7-methoxy-2-methyl-3-phenyl-4-phenylbenzopyranone	973	—	—	—	—	82.57 ± 10.91	—
39	4,5-Dihydro-1H-penten-2-one	899	—	—	—	—	—	136.70 ± 9.13
40	cis,trans-2.3-Dimethylcyclobutanon	749	—	—	27.92 ± 2.51	—	—	—
Aldehydes								
41	Nonyl aldehyde	1,100	68.11 ± 9.29^Ab^	53.64 ± 2.72^Bb^	14.60 ± 3.24^Cb^	125.20 ± 52.75^Aa^	90.33 ± 11.85^Ba^	44.39 ± 20.82^Ca^
42	Phenylacetaldehyde	1,048	—	86.33 ± 10.34^a^	145.17 ± 77.13^a^	—	58.95 ± 10.15^b^	91.48 ± 1.07^b^
43	Hexadecanal	1,822	—	—	—	—	0.230 ± 0.049	—
44	Benzaldehyde	980	44.16 ± 12.97^Cb^	127.27 ± 12.81^Aa^	119.25 ± 12.02^Ba^	88.58 ± 5.66^Ca^	121.10 ± 36.29^Ab^	102.63 ± 23.73^Bb^
45	3-Ethylbenzaldehyde	1,041	—	—	—	4.90 ± 1.02	—	—
46	(E)-2-Octenal	1,057	8.46 ± 2.83^b^	—	—	61.36 ± 16.67^a^	—	—
47	Octanal	1,005	14.12 ± 4.32^b^	21.06 ± 2.69	—	80.05 ± 15.83^a^	—	—
48	Butanedial	994	—	—	—	49.99 ± 0.43	—	—
49	Hexanal	850	269.83 ± 182.61^b^	152.74 ± 28.21	—	861.31 ± 33.34^a^	—	—
50	3-methyl hexanal	941	—	—	—	53.091 ± 33.335	—	—
51	Methyl valeraldehyde	800	—	10.43 ± 2.38^b^	—	—	60.22 ± 7.99^a^	4.48 ± 0.44
52	3-(Methylthio)propionaldehyde	915	—	25.75 ± 1.48^b^	66.25 ± 16.46	—	43.23 ± 17.18^a^	—
53	Benzeneacetaldehyde, a-ethylidene-	1,211	—	—	—	—	—	11.82 ± 3.70
54	Isovaleraldehyde	655	—	—	95.74 ± 5.93	—	—	—
Acids								
55	Isovaleric acid	880	13.58 ± 1.03	—	41.21 ± 4.61	—	—	—
56	Acetic acid glacial	741	30.43 ± 3.00^A^	33.92 ± 1.92^B^	94.47 ± 6.34^Ca^	—	—	71.43 ± 17.08^b^
57	Butyric acid	791	—	12.63 ± 0.90	13.61 ± 2.96	—	—	—
58	Isobutyric acid	758	—	—	5.08 ± 0.67	—	—	—
59	L-CYSTEIC ACID	826	—	65.39 ± 0.75	—	28.56 ± 7.24	—	—
60	2-(2-acetylhydrazide)propionic acid	774	—	—	—	—	12.64 ± 1.83	—
61	Hexanoic acid	980	28.20 ± 1.01	—	—	—	—	—
62	Valeric acid	900	—	—	3.86 ± 1.01	—	—	—
63	2-Methylbutyric acid	895	—	—	34.23 ± 3.82	—	—	—
Esters								
64	Vinyl acetate	776	—	—	—	—	55.64 ± 3.53	19.08 ± 2.17
65	Isobutylamyl sulfite	926	—	5.95 ± 0.39	20.62 ± 2.36^b^	—	—	24.37 ± 2.11^a^
66	Ethyl caprate	1,370	—	—	—	—	—	1.39 ± 0.19
67	Butylpropyl oxalate	893	—	21.38 ± 0.79	—	3.04 ± 0.67	—	—
68	Methyl mercaptan caproate	845	4.34 ± 1.16^b^	—	—	11.53 ± 0.77^a^	—	—
69	Hexanoic acid, ethenylester	768	—	11.38 ± 0.89	—	12.76 ± 1.34	—	—
70	2-Methoxy acetate	835	7.37 ± 0.89^b^	6.11 ± 0.51	—	17.62 ± 0.94^a^	—	—
71	1-Propene, 3-propoxy-	990	—	—	140.58 ± 1.92	—	—	—
Hydrocarbons								
72	Tetradecane	962	—	—	—	101.65 ± 3.98	—	—
73	2,4-Dimethyldecane	733	—	—	—	—	7.82 ± 2.89	—
74	2,2-Dimethylpentane	675	—	12.25 ± 1.29^b^	—	—	38.79 ± 2.78^a^	—
75	2,2,6,6-Tetramethylheptane	763	—	—	16.18 ± 2.71	—	—	—
76	1-Iodotetradecane	872	—	33.62 ± 3.13^a^	—	—	27.59 ± 1.50^b^	—
77	3-Ethylpentane	682	—	—	—	—	21.621.13	—
78	Nitrocyclohexane	951	—	—	—	8.30 ± 2.50	—	—
79	2,2-Dimethylbutane	567	50.76 ± 0.30^b^	—	—	52.79 ± 0.27^a^	—	5.64 ± 0.43
80	3,7-Dimethylnonane	749	—	—	25.67 ± 1.99^b^	33.27 ± 6.60	—	87.65 ± 0.84^a^
81	Pentadecane	853	—	—	—	170.32 ± 1.17	—	—
82	Isopropylcyclobutane	760	—	—	—	18.64 ± 1.96	—	—
83	Hexadecane	868	—	—	—	62.13 ± 22.00	—	—
84	3,3-Dimethylhexane	744	4.47 ± 0.30^b^	—	—	20.32 ± 1.56^a^	11.65 ± 1.06	—
85	2-methyl-Propane	753	—	—	—	16.62 ± 0.48	—	—
86	1,1-Dimethylcyclopentane	673	—	—	—	18.89 ± 1.35	—	6.12 ± 0.23
87	1-Iodononane	1,000	4.87 ± 0.61	—	—	—	13.60 ± 3.01	—
88	3-methyl-Hexane	671	—	—	—	—	54.76 ± 7.56	—
89	3,4,5,6-tetramethyloctane	946	—	—	—	—	180.78 ± 22.10	37.23 ± 2.68
90	4-Methyl-5-propylnonane	851	—	—	—	—	40.59 ± 2.19	—
91	3,6-Dimethyldecane	932	—	—	—	—	—	99.36 ± 1.88
92	Undecane, 3-methyl-	1,171	—	—	10.66 ± 3.02	—	—	13.39 ± 0.84
93	1-Iodododecane	900	—	4.33 ± 1.02	17.54 ± 2.81	—	—	—
94	2-Bromo-1,1,3-trime-thoxypropane	715	2.55 ± 0.39	—	—	—	—	—
95	3-Methyldecane	886	—	—	21.27 ± 1.01	—	—	—
96	3-ethyl-Hexane	770	—	—	39.20 ± 3.46	—	—	—
97	2-Methyl-2-nitropropane	889	—	—	18.87 ± 2.26	—	—	—
98	4-Propylheptane	1,001	—	—	51.71 ± 3.98	—	—	—
Others								
99	Acetylvaleryl	782	—	—	18.07 ± 1.70	6.45 ± 0.84	—	—
100	Dimethyl ether	961	—	—	—	95.78 ± 1.91	—	187.06 ± 33.31
101	Sulfur dioxide	880	12.41 ± 21.49^b^	—	70.53 ± 3.77^a^	59.33 ± 1.15^a^	14.84 ± 2.02	39.50 ± 7.72^b^
102	2,6-Dimethylpyrazine	819	—	—	15.55 ± 3.21^b^	—	—	24.70 ± 4.23^a^
103	4-Hydroxytoluene	1,098	—	—	—	—	—	43.02 ± 15.33
104	2,3,5,6-Tetramethylpyrazine	1,087	—	—	5.42 ± 0.85^b^	—	—	39.55 ± 14.44^a^
105	2-furanocarboxylic acid, tetrahydro-3-methyl-5-oxo	814	6.40 ± 1.01	—	—	39.67 ± 3.77	—	—
106	N-Methyl-N-(1-methyl-2-phenylethyl)-O-2-furanmethylamine	791	3.84 ± 0.57	8.31 ± 0.71	—	—	—	—
107	N,N,N′-Trimethylethylenediamine	1,107	—	—	—	—	—	244.43 ± 75.36
108	2,3,5-Trimethylpyrazine	1,001	—	—	—	—	—	133.79 ± 47.82
109	4-Methyl-2,4-bis(p-hydroxyphenyl)5-penten-1-ene,2TMS derivative	1,045	—	68.91 ± 2.20	3.52 ± 0.08^b^	—	—	43.36 ± 3.01^a^
110	Butane, 1-isocyano-	725	—	—	—	—	—	9.99 ± 1.52
111	Butanimidamide	768	16.61 ± 1.72	—	—	—	—	47.54 ± 2.60
112	1,6-Anhydro-3,4-dideoxy-β-D-glucose hexapyranose	694	—	—	7.11 ± 5.00	—	—	—

MY represents low-altitude ham (1,600 m above sea level), MP represents high-altitude ham (2,544 m above sea level), MY0, MY1, and MY2 denote Mianning hams fermented for 0, 1, and 2 years at low altitude, respectively, and MP0, MP1, and MP3 denote coronation hams fermented for 0, 1, and 2 years at high altitude, respectively. Different uppercase letters (A, B, C) denote the variation between different years at the same altitude (*p* < 0.05), and different lowercase letters (a, b) denote the variation between different altitudes at the same year (*p* < 0.05), “–” indicates that the system is not detected. KI-retention indices.

The flavor formation of dry-cured ham is influenced by alcohols, which are one of the important volatile compounds. The formation of alcohols is closely related to lipid oxidation (Shi et al., 2019). Alcohol is a crucial contributor to the characteristic flavor of dry cured meat products. The types of alcohol in MP were more abundant than those in MY (*p* < 0.05). 1-Hexanol decreased first and then increased in MP, and the content of MP1 was the highest (172.18 ± 15.46 μg/kg). 1-octene-3-ol was produced in ham fermented for 0, 1 and 2 years at the two altitudes, and the absolute content of 1-octene-3-ol in MY0 and MP0 at the same altitude was significantly higher than that in the other two fermentation years, being 170.85 ± 7.99 μg/kg and 309.66 ± 115.59 μg/kg, respectively. It was found that 1-octene-3-ol was also used as a characteristic flavor substance in Dahe black pig ham (Wei et al., 2023).

Ketones are usually volatile compounds formed through lipid oxidation, Maillard reaction, and thiamine degradation and significantly impact food flavor (Grebenteuch et al., 2021). Volatile carbonyl compounds, including ketones, were studied during the maturation of Iberian ham; the study found that ketones occupy a place in the volatile flavor substances of ham and significantly impact the overall flavor of Iberian ham. These ketones may come from lipid oxidation and microbial metabolism and are usually associated with ham’s creamy, fruity, and ripe flavor (Narváez-Rivas et al., 2014). 2-Nonanone and 2-Heptanone are the main ketones of MY and MP. At the same altitude, the content of 2-Nonanone increases continuously with the extension of the processing year. The content of 2-heptanone in MP1 (423.17 ± 105.39) was significantly higher than that in MY1 (377.30 ± 5.75) (*p* < 0.05). In Jinhua ham, 2-heptanone is one of the primary essential flavor substances, which makes an important contribution to the flavor of ham (Liu et al., 2020).

Nonyl aldehyde and Benzaldehyde are two aldehydes with higher content produced in MY and MP, and the content of Nonyl aldehyde in MP is higher than that of MY. At the same altitude, the content of Nonyl aldehyde significantly decreases with the extension of the fermentation year (*p* < 0.05). The absolute content of Benzaldehyde in MY1 and MP1 was significantly higher than that in the other 2 years (*p* < 0.05), which was 127.27 ± 12.81 μg/kg and 121.10 ± 36.28 μg/kg, respectively. Wang L. et al. (2021) found that the flavor of Anfu ham is closely linked to the climate of its region of origin, and Nonyl aldehyde is also a key flavor substance, which is consistent with the results of this study. At the same altitude, the content of Benzaldehyde in ham fermented for 1 year was significantly higher than in the other 2 years (*p* < 0.05).

Acid compounds are important volatile components in dry-cured ham. They may be derived from oxidative degradation of glycerol and phospholipids, Maillard reaction, or high-temperature oxidation of aldehydes (Wang et al., 2018). MY acids were significantly higher than MP (*p* < 0.05), which may be due to the generally higher ambient temperature at low altitudes, which accelerated the fat and protein degradation process in ham, generating more acids. The absolute content of acetic acid was the highest in MY, and the content of acetic acid increased significantly with the increase of fermentation time (*p* < 0.05). Wang T. et al. (2021) found that acetic acid in 150d fermented Dahe black pig ham was also the primary volatile flavor substance.

Esters are usually formed by the reaction of alcohols and carboxylic acids under the catalysis of esterification enzymes. The absolute content of esters in MY1 was significantly higher than that in MP1 (*p* < 0.05). Hydrocarbons and other volatile compounds have no significant effect on fragrance due to their high odor threshold (Marušić Radovčić et al., 2021). The results showed that the content of MP hydrocarbons and other substances was significantly higher than that of MY. There were more alkanes in the early stage of MP fermentation (*p* < 0.05), which may be because the fermentation environment, such as temperature and humidity, had significant effects on the lipid oxidation rate in the early stage of fermentation. The environmental conditions in the early stage of fermentation may be more favorable to the occurrence of lipid oxidation reaction, thus increasing the formation of alkanes (Mai et al., 2024).

To further understand the difference in the contribution of volatile compounds to the overall flavor characteristics of MY and MP, the OAV of each compound was calculated based on the absolute content of each volatile flavor substance and the odor threshold (OTV) in previous literature (Wang et al., 2024). It is generally believed that when OAV ≥ 1, the contribution of volatile flavor compounds to the overall flavor is more significant (Huang et al., 2024). The results are shown in Table 3. According to the OAV value, a total of 15 major flavor substances were selected and identified as affecting the overall flavor characteristics of Mianning ham in the two altitude areas, including 5 alcohols, 4 ketones and 6 aldehydes, among which 1-octene-3-alcohol and Nonyl aldehyde contributed more to the flavor of Mianning ham in the two altitude areas. Related studies have shown that 1-octene-3-ol is a compound with a mushroom aroma, and cooking methods such as steaming and cooking may increase the content of 1-octene-3-ol in ham, thereby improving its flavor. In addition, the concentration of 1-octen-3-ol may vary in different parts of the ham and at different stages of maturation, which may affect the overall flavor profile of hams (Inamdar et al., 2020). At the same altitude, the flavor of 1-octene-3-ol decreased significantly with the extension of fermentation (*p* < 0.05). Nonyl aldehyde has a solid fat taste, and it is also selected as one of the main aroma substances of Iberian ham and has a significant contribution to the meat aroma, fragrance, and fat aroma of ham, which is similar to the results of this study (García-González et al., 2013). During processing, the aroma of nonanal was significantly reduced (*p* < 0.05), with nonanal contributing the most to the aroma in 0 year hams at both altitudes. N-octaldehyde is a sharp and powerful fat wax fragrance, which is more intense in MP0, and also contributes to MY0 and MY1. The content of 3-(Methylthio) propionaldehyde is higher in MY2 and MP1. Liu et al. (2024) provided an overview of 3-(Methylthio) propionaldehyde, including its use in the food industry mainly as a flavor enhancer in meat products. This compound has been used to enhance the flavor of food products due to its boiled potato-like odor.

**Table 3:** Main volatile flavor compounds of Mianning ham at different altitudes (OAV > 1).

Volatile compound	OTV (μg/kg)	MY			MP		
		MY0	MY1	MY2	MP0	MP1	MP2
Phenethyl alcohol	45	–	–	–	–	–	4.40
1-Octen-3-ol	2	85.43	21.66	5.84	154.83	22.05	8.10
1-Octanol	10	5.55	–	–	7.35	–	–
1-Hydroxy	4.8	2.45	–	–	5.41	–	–
1-Butanol	100	–	2.09	–	–	–	–
2-Nonanone	25	–	1.38	3.93	–	2.59	4.11
3-Hydroxy-2-butanone	40	1.65	–	–	3.44	–	–
2-Heptanone	70	–	5.39	3.79	–	6.05	5.24
2-Octanone	50	–	–	–	–	–	1.28
Nonyl aldehyde	3.5	19.46	15.32	4.17	35.77	25.81	12.68
Phenylacetaldehyde	9	–	9.52	16.13	–	6.55	10.16
Benzaldehyde	50	–	2.55	2.38	1.77	2.42	2.05
Octanal	0.1	141.15	210.62	–	800.48	–	–
Hexanal	7.5	35.97	20.37	–	114.84	–	–
3-(Methylthio)propionaldehyde	0.04	–	643.85	1,656.18	–	1,080.78	–

Odor threshold (OTV) and Odor activity (OAV) represent odor threshold and odor activity value, respectively. “–” indicates no.

### 3.3 Analysis of microbial diversity

#### 3.3.1 Analysis of bacterial diversity

The alpha-diversity of bacteria in the two elevations is shown in Table 4. In MY, the Chao1, Shannon and Simpson indices of MY1 are the highest, while in MP, the Chao1, Shannon and Simpson indices of MP2 are the highest. The results showed that there were significant differences in bacterial species between the two elevations (*p* < 0.05).

**Table 4:** 16S RDNA-α diversity index of bacteria from Mianning ham at different altitudes.

Samples	Observed species	Chao1	Shannon	Simpson	Coverage
MY0	287.00 ± 23.26	387.05 ± 18.41	1.66 ± 0.05	0.66 ± 0.06	0.99 ± 0.00
MY1	711.33 ± 33.62	775.61 ± 32.18	3.82 ± 0.71	0.89 ± 0.08	0.99 ± 0.00
MY2	256.00 ± 26.90	282.08 ± 19.11	1.57 ± 0.03	0.49 ± 0.13	0.99 ± 0.00
MP0	342.00 ± 26.02	412.80 ± 39.07	2.46 ± 0.02	0.79 ± 0.09	0.99 ± 0.00
MP1	305.33 ± 24.68	375.01 ± 40.94	2.29 ± 0.05	0.81 ± 0.01	0.99 ± 0.00
MP2	320.67 ± 45.65	352.53 ± 44.34	3.06 ± 0.66	0.80 ± 0.13	0.99 ± 0.00

**Figure 1:**
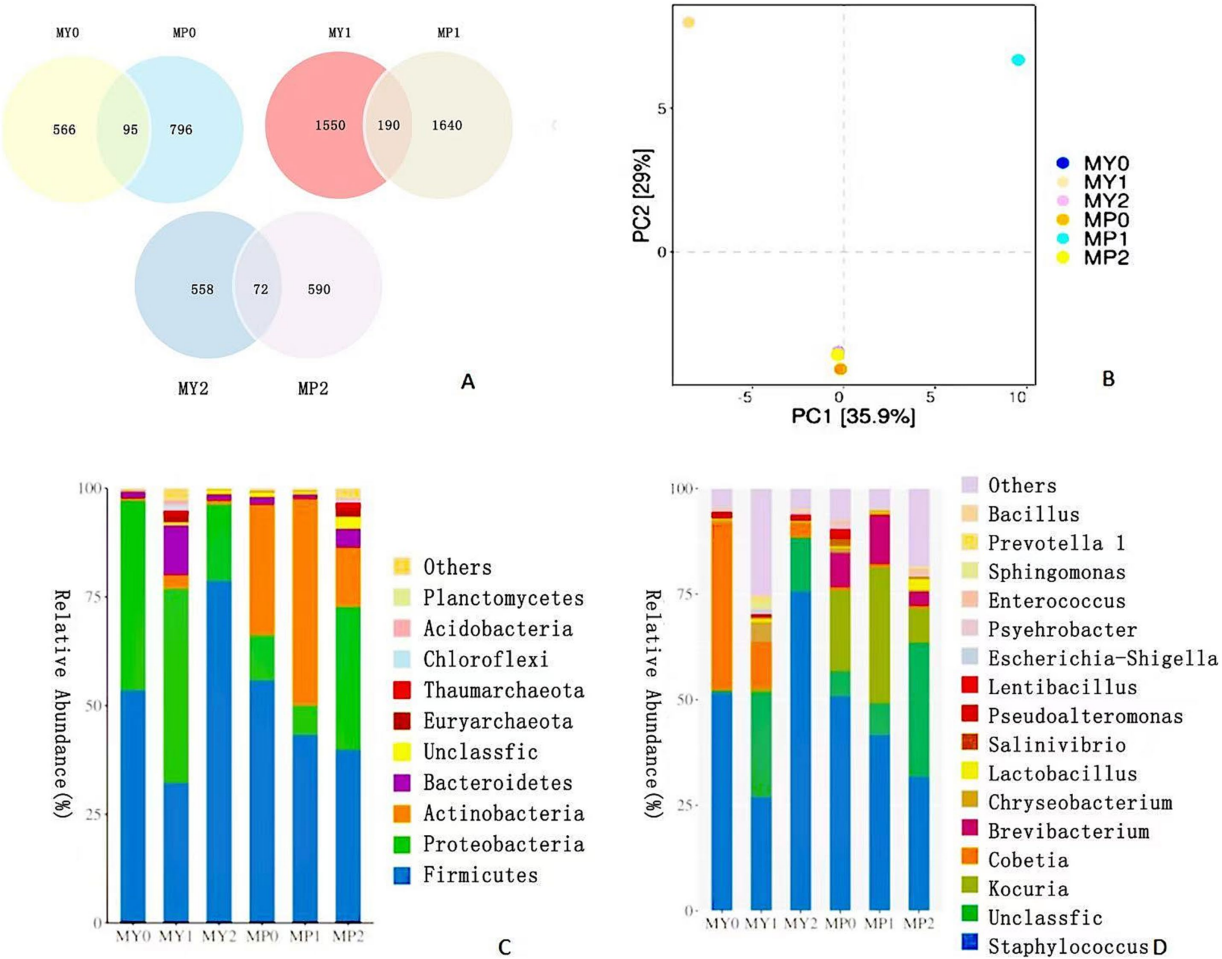
Venn diagram **(A)** of bacterial diversity of Mianning ham at different altitudes. The numbers in different compartments represent the number of bacteria unique or common to ham at different altitudes in the same fermentation year. OTU based bacterial principal component analysis score chart **(B)**; Bacterial colony maps at phylum level **(C)** and genus level **(D)**.

According to the cluster analysis results, the Venn diagram (Figure 1A) was used to compare the individual and common OTUs of 0, 1 and 2 year fermented ham at two altitudes, respectively. There were 95, 190 and 72 common OTUs in 0, 1 and 2 year fermented ham at different altitudes, respectively. It can be seen from the figure that MP bacterium OTUs are higher than MY (*p* < 0.05), which is closely related to the climatic conditions in the high altitude area, and the specific factors need to be further studied. It is worth noting that the number of OTUs of ham fermented for 2 years is the largest. During the fermentation process, the number of OTUs of Mianning ham in the two elevations shows a trend of first increasing and then decreasing, which is significantly related to the salt content of the ham. As mentioned above, the salt content increases significantly with the extension of the fermentation time of ham, so the increase of salt inhibits the growth and reproduction of bacteria.

As shown in Figure 1B, due to the effect of fermentation time, the positions of 0 and 2 years of low-altitude ham overlap on the PC1 and PC2 axes, indicating that their bacterial community structure is very similar. With the increase of fermentation time, MY1 increased on the PC2 axis, indicating that the bacterial community changed at the 1 year stage. Similarly, the close distance between 0 and 2 years of high altitude ham indicated that the bacterial communities in these two stages did not change much, indicating that the bacterial composition of ham at the early stage of fermentation and 2 years of fermentation was similar. In the comparison between low and high altitudes, the effect of altitude on bacterial community showed that MY and MP showed apparent differences in the 1 year PCA diagram (*p* < 0.05). However, there were some similarities in the fermentation stages of 0 and 2 years, significantly the close overlap between MY2 and MP2, indicating that the bacterial community structure of low-altitude and high-altitude ham began to be similar at 2 years of fermentation. This is consistent with the possible convergence of microbial communities during fermentation, and the differences between bacterial communities in different regions may gradually decrease over time. MY0 was overlapped with MY2, indicating that the bacterial communities of fermented 0 year and 2 year ham at low altitudes were similar. In summary, the bacterial communities of ham at low altitude and high altitude changed gradually with the fermentation time. There was a significant change in the first year of fermentation (*p* < 0.05), and there was overlap in the fermentation stage of 0 years and 2 years. After 2 years of fermentation, the bacterial community structure of ham at different elevations began to resemble, suggesting that the influence of the long-term fermentation process on the bacterial community may exceed the influence of altitude.

In order to further understand the bacterial flora structure in the two elevations, high-throughput sequencing technology was used to analyze the bacterial phyla (Figure 1C) and bacterial genera (Figure 1D) in the fermented 0 year, 1 year and 2 year ham in the two altitudes. At the phylum level, 45 phylum were identified, among which Firmicutes, Proteobacteria and Actinobacteria ranked in the top three dominant phyla, which were similar to the dominant phylum in Nuodeng ham (Li et al., 2024c). Firmicutes are the dominant bacteria in ham at two altitudes, while Proteobacteria are abundant in ham at 0, 1 and 2 years of fermentation at low altitudes, with relative abundances of 43.70, 44.69 and 17.71%, respectively. Actinobacteria is the predominant bacteria compared with MP in MY. The relative abundance of actinomycetes in 0 year, 1 year and 2 year fermented ham was 29.92, 47.45 and 13.51%, respectively, indicating apparent differences between the two elevations.

At the genus level, the 20 microorganisms high relative abundance include *Staphylococcus*, *Cobetia*, and *Kocuria*. *Staphylococcus* is a dominant bacterium common to ham at two elevations and in dry-cured ham. It can promote the degradation of fat and protein in ham, contribute to the biosynthesis of volatile flavor compounds, and play an essential role in the color development of ham (Li et al., 2023). In addition, the *Cobetia* also occupies an important position in MY0 and is competitive with *Staphylococcus*. *Kocuria* and *Brevibacterium* had the highest relative abundance in MP1, with 32.19% (*p* < 0.05) and 12.77% (*p* < 0.05), respectively. *Kocuria*, as a particular genus of bacteria at MP, can competitively inhibit the growth of undesirable microorganisms and reduce the generation of harmful substances such as amines, thus improving the edible safety of ham (Shi et al., 2021). This is also a unique advantage of MP compared with MY.

#### 3.3.2 Analysis of fungal diversity

The fungal alpha-diversity in the two altitude areas is shown in Table 5. Shannon in the two altitude areas shows a trend of first decreasing and then increasing, among which the Chao1, Shannon and Simpson indices in MY0 are the highest, which are 587.47, 3.91 and 0.91 respectively, indicating that MY0 has the highest fungal community richness. The Shannon index of MP1 was significantly higher than that of MY1 (*p* < 0.05).

**Table 5:** Diversity index of fungi 1TS of Mianning ham at different altitudes.

Samples	Observed species	Chao1	Shannon	Simpson	Coverage
MY0	557.67 ± 33.52	587.47 ± 17.73	3.91 ± 0.63	0.91 ± 0.10	0.99 ± 0.00
MY1	388.33 ± 27.76	415.02 ± 22.20	1.59 ± 0.18	0.4 ± 0.04	0.99 ± 0.00
MY2	360.67 ± 23.09	373.56 ± 24.44	2.38 ± 0.15	0.66 ± 0.03	0.99 ± 0.00
MP0	527 ± 19.08	558.67 ± 17.52	3.17 ± 0.14	0.8 ± 0.07	0.99 ± 0.00
MP1	394 ± 45.17	419.26 ± 14.25	2.26 ± 0.60	0.62 ± 0.12	0.99 ± 0.00
MP2	367 ± 19.08	387.09 ± 28.97	2.46 ± 0.12	0.74 ± 0.04	0.99 ± 0.00

**Figure 2:**
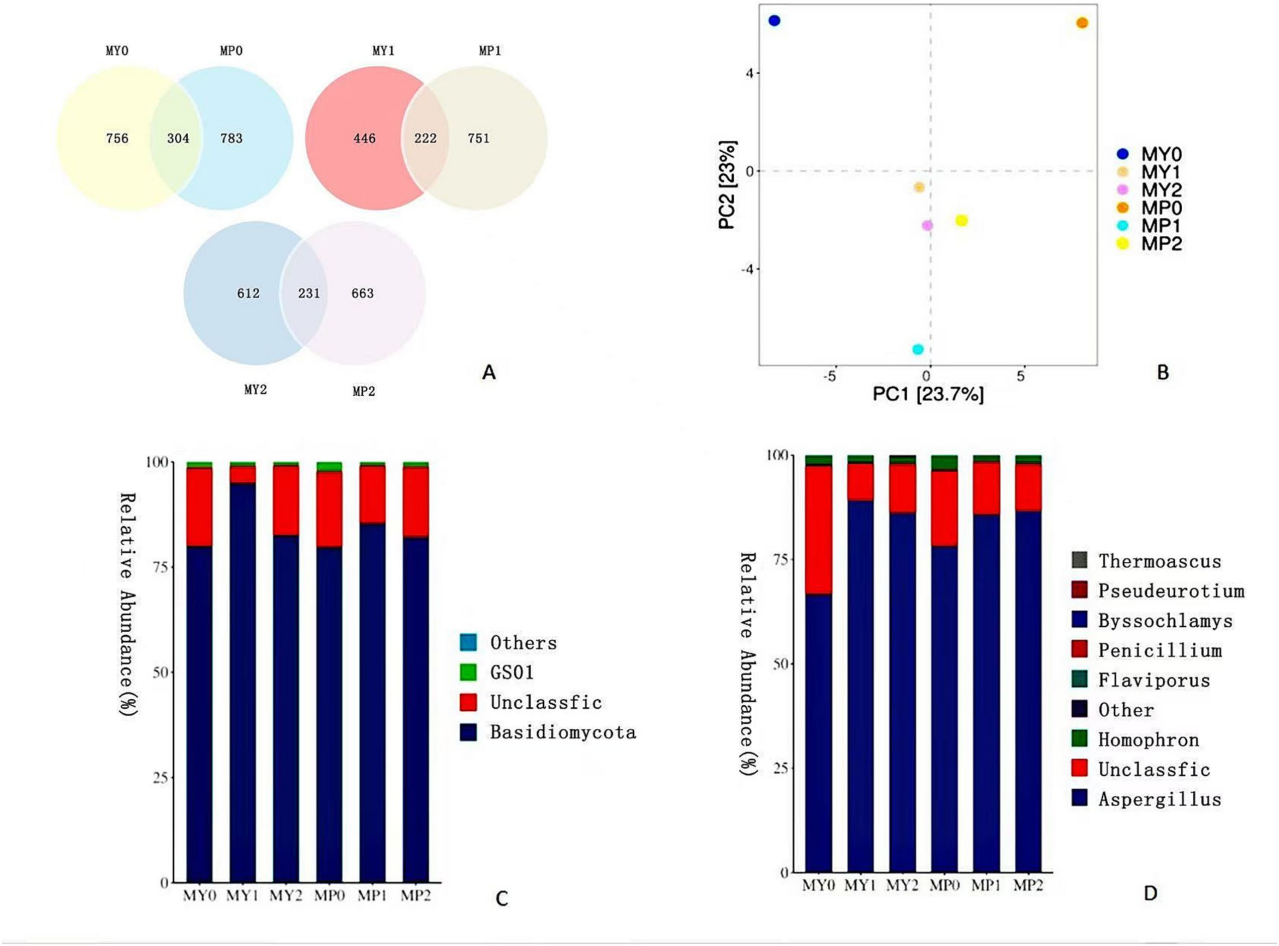
Venn diagram **(A)** of fungal diversity of Mianning ham at different altitudes. The numbers in different compartments represent the number of fungi unique or common to ham at different altitudes in the same fermentation year. OTU based fungal principal component analysis score chart **(B)**; Fungal colony maps at phylum level **(C)** and genus level **(D)**.

According to the cluster analysis results, a Venn diagram (Figure 2A) was used to compare the individual and common OTUs of 0, 1 and 2 year fermented ham at two altitudes, respectively. There were 304, 222 and 231 OTUs in 0, 1 and 2 year fermented ham at different altitudes, respectively. Among them, MP fungus OTUs had the highest water content during fermentation, and the water content was closely related to fungal growth, consistent with previous studies on Xuanwei ham (Li et al., 2024a). Therefore, the results showed that the fungal flora diversity of MP was higher than that of MY.

As can be seen from Figure 2B, MY is mainly distributed in the left region of the figure, and MY1 and MY2 are close to each other on the PC1 and PC2 axes, indicating that the fungal community structure of 1 year and 2 year fermented Mianning ham was similar. MY0 is located in the upper left corner, indicating significant changes in the fungal community at the beginning of fermentation. The distribution of MP is mainly concentrated in the right area of the figure. The distribution of MP0, MP1, and MP2 is relatively dispersed. The location difference is noticeable; mainly, MP0 is located in the upper right corner, indicating that the fungal community changes significantly in the early stage of high-altitude ham fermentation, and there are differences in the fungal community between 1 year and 2 year ham (*p* < 0.05). The community differences between MY and MP were evident on the PCA map. In particular, the distance between MY0 and MP0 was the longest, indicating that the fungal communities of the two kinds of ham significantly differed in the year 0 fermentation, followed by the distance between MY1 and MP1. The changes of MY and MP fungal communities at different stages of fermentation showed different evolution trends. MY changed little in the latter two stages, while MP showed a more remarkable dynamic change (*p* < 0.05), that is, the fungal community of MP changed more significantly with the increase of fermentation time.

In order to further understand the fungal flora structure of ham from low altitude and high altitude areas, the colony composition of Mianning ham from two altitude areas was analyzed at the phylum level (Figure 2C) and genus level (Figure 2D). At the phylum level, the dominant phylum shared by MY and MP was Basidiomycota, with the relative abundance of MY0, MY1 and MY2 being 79.84, 94.82 and 82.47%, respectively, and the relative abundance of MP0, MP1 and MP2 being 79.64, 85.28 and 82.04%, respectively. There was no significant difference in the relative abundance of Basidiomycota between 0 and 2 years of fermented ham at two altitudes. However, the relative abundance of 1 year fermented ham was significantly higher than that of the other 2 years (*p* < 0.05). It is worth noting that the fungal dominant phyla of Nuodeng ham and Mianning ham are similar, both of which are Basidiomycota. This indicates that there may be certain similarities in the succession of fungal communities between the two hams during the fermentation process, and Basidiomycota play an important role in both of them, which may have similar influence mechanisms on the formation of flavor and quality of ham (Li et al., 2024b). Related studies have shown that during the fermentation process, Basidiomycetes fungi can secrete a variety of enzymes, such as protease, amylase, and lipase, which help to decompose ample molecular nutrients in ham and convert them into small molecules that are easier for human digestion and absorption (Akaniro et al., 2023). In addition, these enzymes promote the formation of flavor substances in the ham, such as esters, acids, and aldehydes, which are essential for the final flavor of the ham.

At the genus level, the dominant bacterium shared by MY and MP is *Aspergillus*, which is similar to Jinhua ham (Deng et al., 2022). *Aspergillus* plays an essential role in fermented food (Huang et al., 2024). In MY0, the relative abundance of Aspergillus was the lowest, accounting for only 68.85%. The growth and reproduction of *Aspergillus* require a certain period of time for accumulation to reach a sufficient quantity, so as to exert its role in the fermentation process (Son et al., 2023). However, the fermentation time of 0 year ham in the two elevations was short, so the amount of *Aspergillus* could accumulate was small. The relative abundance of MP1 *Aspergillus* is significantly higher than that of MP0 (*p* < 0.05). There is no significant difference in the relative abundance of *Aspergillus* between 1 year and 2 year fermented ham. It is worth noting that MY1 has the highest water content, which provides an excellent survival advantage for *Aspergillus*.

### 3.4 Correlation analysis between microorganisms and key flavor substances

**Figure 3:**
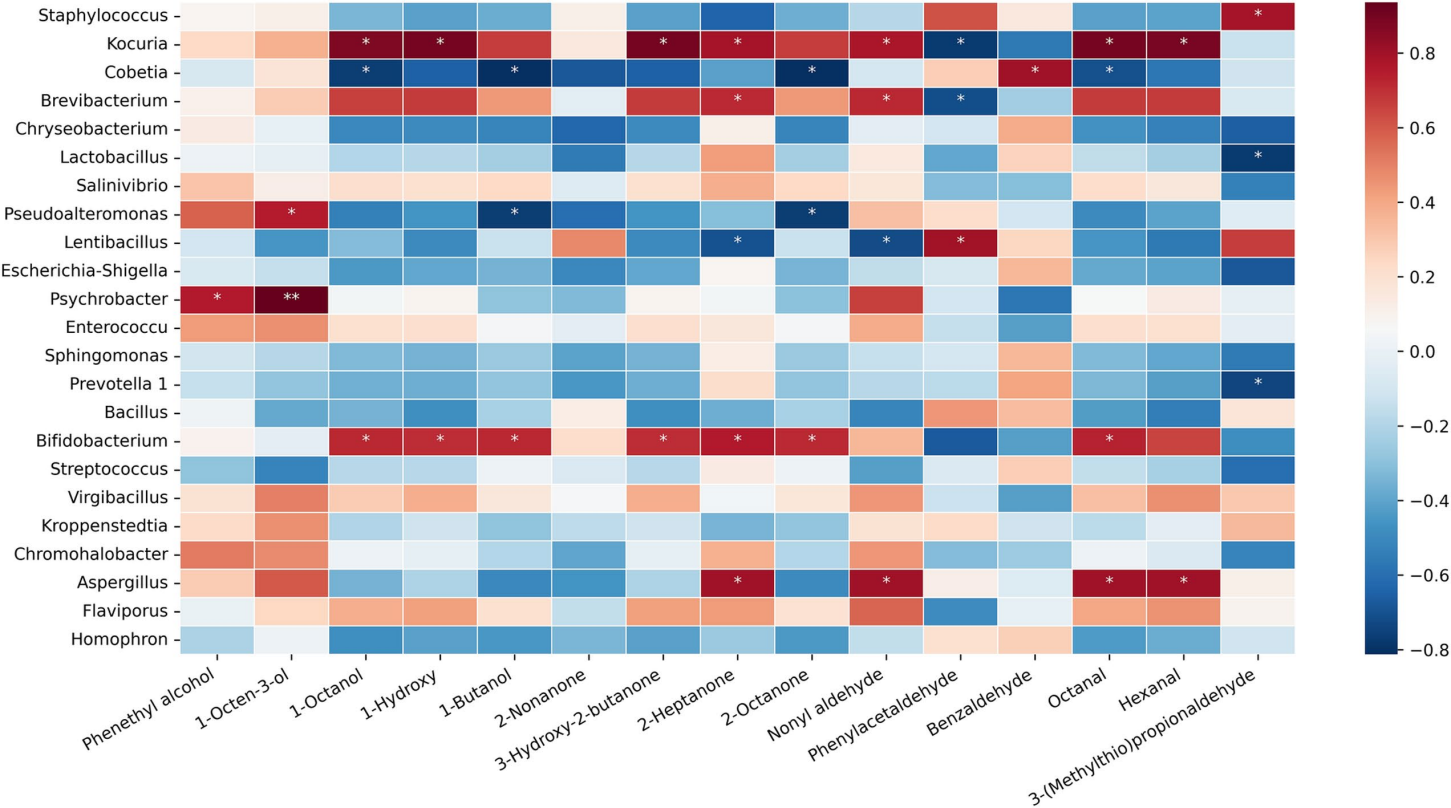
Heat map of correlation analysis between dominant microbial genera and key flavor compounds of Mianning ham in two elevations. In the correlation heat map, the first 20 rows show the correlation between dominant bacteria genera and key volatile compounds, and the last three rows show the correlation between dominant fungi genera and key volatile compounds; *Significant correlation; **Extremely significant correlation.

The ham’s flavor formation is mainly achieved through the decomposition and oxidation of protein and fat. Microorganisms secreted endogenous henzymes to promote fatty hydrolysis during the ripening process of dry-cured ham. They participated in proteolysis to promote protein degradation and produce polypeptides and free amino acids Zhu et al. (2022). These compounds act as precursors to a large number of flavor substances, thus giving the product a unique flavor. According to the analysis of ham’s dominant microbial genera and essential volatile flavor substances at different altitudes, the thermal aggregation diagram of microbial genera and essential volatile flavor substances was constructed, as shown in Figure 3. *Staphylococcus* had a significant positive correlation with 3-(Methylthio) propionaldehyde (*p* < 0.05), and *Staphylococcus* has the highest content of MY2. Therefore, *Staphylococcus* promotes the formation of MY2’s unique flavor. *Cobetia* is the dominant bacterium of MY. It was negatively correlated with 1-Octanol, 1-Butanol, 2-Octanone, and Octanal (*p* < 0.05) but positively correlated with Benzaldehyde (*p* < 0.05). *Cobetia* has the potential to reduce the flavor diversity of MY. There was a significant positive correlation between *Kocuria* and 1-Octanol, Heptanol, 1-butanol, 2-Heptanone, 3-Hydroxy-2-butanone, Octanal, and Hexanal (*p* < 0.05). Kocuria promoted the formation of seven major flavor substances in MP. Notably, the content of Octanal in MP0 is higher., and the contribution of *Kocuria* to the formation of this flavor is more outstanding. *Psychrobacter* showed a significant positive correlation with 1-octene-3-ol (0.05 > *p* > 0.01), but unfortunately, it is low in MP and may not have a noticeable effect on flavor. *Bifidobacterium* was positively correlated with most alcohols and ketones (*p* < 0.05), which positively affected ham’s flavor formation in the two elevations. There was no significant difference between the dominant fungi genera in ham from the two elevations, and *Aspergillus* was positively correlated with 2-Heptanone, Hexanal, Nonyl aldehyde, and Octanal (*p* < 0.05), which was similar to Jinhua ham (Deng et al., 2022). Therefore, *Aspergillus* may be necessary for producing essential flavor compounds in ham from the two elevations.

## 4 Conclusion

The results showed significant differences in physicochemical properties, volatile flavor substances and microbial diversity of ham between the two altitudes. In addition to salt content, other physical and chemical indexes of high-altitude ham are higher than those of low-altitude ham. With the increase of fermentation years, the pH, malondialdehyde content, nitrite content, chloride content, a* and b* of ham at the same altitude gradually increased. The moisture content, aw, and L*, showed a decreasing trend. A total of 65 volatile compounds were detected in ham at low altitudes, and 91 volatile compounds were detected in ham at high altitudes. 1-octene-3-ol and Nonyl aldehyde were the most important contributors to the maturation of Mianning ham. Octanal and 3-(Methylthio)propionaldehyde give the ham of the two elevations its characteristic fat and cooked potato flavor, respectively. Firmicutes, basidiomycetes, *Staphylococcus* and *Aspergillus* are the common dominant microbial genera in the two regions. The dominant bacterial genus of ham at low altitudes is *Cobetia*, while the dominant bacterial genus at high altitudes is *Kocuria*. *Cobetia* promoted the formation of Benzaldehyde in ham at low altitudes, while *Kocuria* promoted the formation of seven primary flavor substances in ham at high altitudes, *Aspergillus* is closely related to aldehydes. This study not only provides a reference for the difference in quality characteristics of ham at different altitudes, but also provides theoretical support for the development of the ham industry and the protection of geographical indication products.

## Data Availability

The original contributions presented in the study are included in the article/supplementary material, further inquiries can be directed to the corresponding author.
